# Machine Learning–Driven Integration of Cancer Cell Phenotypes Predicts Cisplatin Sensitivity

**DOI:** 10.1002/cam4.71373

**Published:** 2025-11-20

**Authors:** Haruki Ujiie, Tomoko Sakyo, Konomi Oya, Yuto Sugawara, Miyu Ota, Honami Yonezawa, Naoyuki Nishiya

**Affiliations:** ^1^ Department of Pharmacy Iwate Medical University Hospital Shiwa‐gun Iwate Japan; ^2^ Division of Integrated Information for Pharmaceutical Sciences, Department of Clinical Pharmacy, School of Pharmacy Iwate Medical University Shiwa‐gun Iwate Japan; ^3^ Division of Health Chemistry, Department of Clinical Pharmaceutical Sciences, School of Pharmacy Iwate Medical University Shiwa‐gun Iwate Japan

**Keywords:** artificial intelligence, biomarkers, efficacy, lung cancer, RNA‐seq

## Abstract

**Background:**

Precision medicine has personalized anticancer therapies and has been considered standard practice. Although current cancer genomic profiling tests are powerful tools to predict the efficacy of molecular targeted drugs or immune checkpoint inhibitors, they are not readily applicable for classical anticancer agents. In this study, we report a novel concept of phenotype‐based classification using machine learning analysis of gene expression patterns to predict the effectiveness of anticancer agents.

**Methods:**

Hierarchical clustering of IC_50_ values distinguished cisplatin‐sensitive and resistant cell lines. Differentially expressed gene (DEG) analysis and SHAP value‐based machine learning identified 26 key genes, and the cisplatin sensitivity predictor using 26 genes (CSP26G) model was developed.

**Results:**

Cisplatin‐resistant A549CR cells experimentally confirmed the external validity of the CSP26G model. The model also classified patients with non‐small cell lung cancer in The Cancer Genome Atlas (TCGA) clinical database into cisplatin‐sensitive and cisplatin‐resistant groups. The predicted sensitive group showed significantly longer survival than the predicted resistant group. Furthermore, CSP26G predicts not only cisplatin efficacy but also responsiveness to other DNA‐damaging agents.

**Conclusion:**

These findings indicate that the sensitivity prediction model constructed through the integration of DEG and machine learning analyses can forecast drug sensitivity, thereby contributing to the advancement of effective and personalized precision medicine in classical chemotherapies.

## Graphical abstract

The CSP26G model, a predictor of cisplatin sensitivity, was developed using machine learning to integrate cancer cell phenotypes. This model accurately predicted sensitivity in vitro and the prognosis of patients with non‐small cell lung cancer.

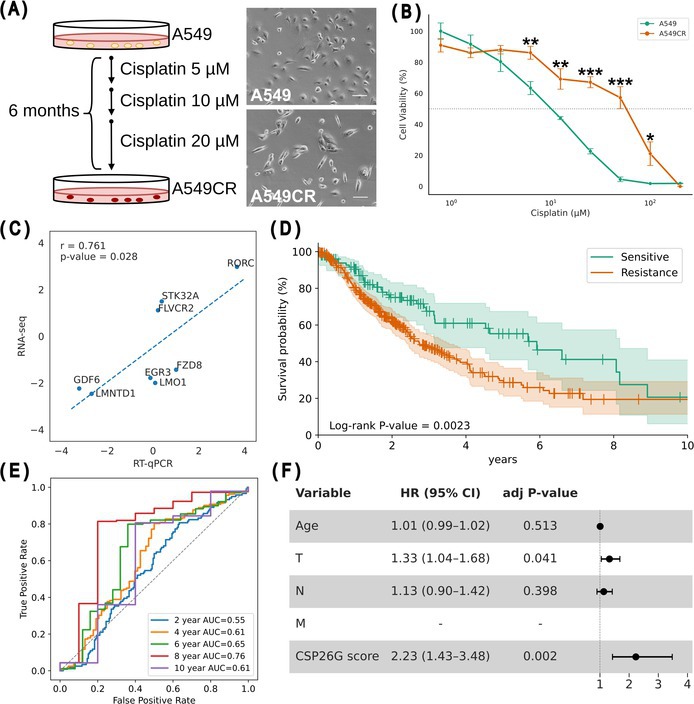

**Abbreviations**
A549CR: A549 cisplatin resistant
AUC: area under the curve
cDNA: complementary DNA
CI: confidence interval
COSMIC: catalog of somatic mutations in cancer
DEG: differentially expressed gene
DepMap: cancer dependency map
FC: fold change
GDSC: genomics of drug sensitivity in cancer
IC_50_
: 50% inhibitory concentration
NSCLC: non‐small‐cell lung cancer
PRISM: profiling relative inhibition simultaneously in mixtures
RFECV: recursive feature elimination with cross‐validation
RNA‐seq: RNA‐sequencing
ROC: receiver operating characteristics
RT‐qPCR: reverse transcription‐quantitative polymerase chain reaction
SHAP: Shapley additive explanations
TCGA: The Cancer Genome Atlas

## 1 Introduction

Cisplatin, a conventional but widely used anticancer agent, induces apoptosis by disrupting the DNA double‐helical structure [1]. Currently, it is estimated to be used in the treatment of approximately 50% of patients with cancer [2] and has demonstrated broad efficacy against various solid tumors, including lung and esophageal cancers [3].

Precision medicine, which is grounded in biomarker analyses derived from each patient's genetic background or tumor molecular profile, has become widely implemented [4]. Precision medicine assures the optimization of treatment for each patient [5] and has been applied clinically through cancer genomic profiling tests. However, precision medicine therapeutics are largely based on highly specific molecular targeted agents such as EGFR and BCR‐ABL1 inhibitors [6, 7, 8]. Cytotoxic anticancer agents which exhibit a wide target spectrum, such as cisplatin, are not typically applicable for precision medicine when predicting drug sensitivity of tumors. On the other hand, assessing germline genetic variation in genes such as *TPMT* and *ACYP2* may be useful for mitigating the toxicity of cisplatin [9, 10]. Therefore, despite cisplatin still being administered to a large number of patients with cancer as a first‐line therapy, a considerable proportion of them receive it based on empirical evidence rather than biomarkers supporting its efficacy. This population may miss the opportunity to receive more effective treatments than the current standard regimen and may even face the risk of nephrotoxicity [3, 11] or ototoxicity [3, 12] without antitumor benefits. Therefore, expanding the framework of precision medicine to classic cytotoxic anticancer agents including cisplatin should optimize treatment selection for a large population.

Recently, the development and availability of large‐scale datasets have expanded and afforded significant advancements in artificial intelligence and machine learning methodologies across diverse scientific disciplines [13]. Furthermore, various omics analyses have been increasingly incorporated into clinical laboratories, thereby raising the demand for machine learning‐based data processing [14]. In the medical field, different applications, such as diagnosis support and evaluation of treatment effects, have been constructed by analyzing diverse data such as histopathological images [15], clinical test data [16], and gene expression data [17].

In this study, a set of 55 feature genes was identified to predict cisplatin sensitivities using integrated analysis of machine learning and bulk RNA‐sequencing (RNA‐sq) of cancer cell lines. Further, machine learning narrowed to 26 biomarker genes and developed a prediction model, cisplatin sensitivity predictor using 26 genes (CSP26G). Furthermore, CSP26G classified non‐small cell lung cancer (NSCLC) patients in The Cancer Genome Atlas (TCGA) database into cisplatin‐sensitive and cisplatin‐resistant groups, and accurately predicted their clinical outcomes. In addition, although CSP26G was originally developed for cisplatin, its sensitivity prediction also significantly correlated with responses to other DNA‐damaging anticancer agents including topoisomerase I inhibitors. These findings suggest that CSP26G could be applicable to DNA‐damaging anticancer agents other than cisplatin, contributing to the advancement of effective and individualized precision medicine with classical anticancer agents.

## 2 Materials and Methods

### 2.1 Definition of Cell Groups Resistant and Sensitive to Cisplatin Through the Matching of Public Databases

**FIGURE 1:**
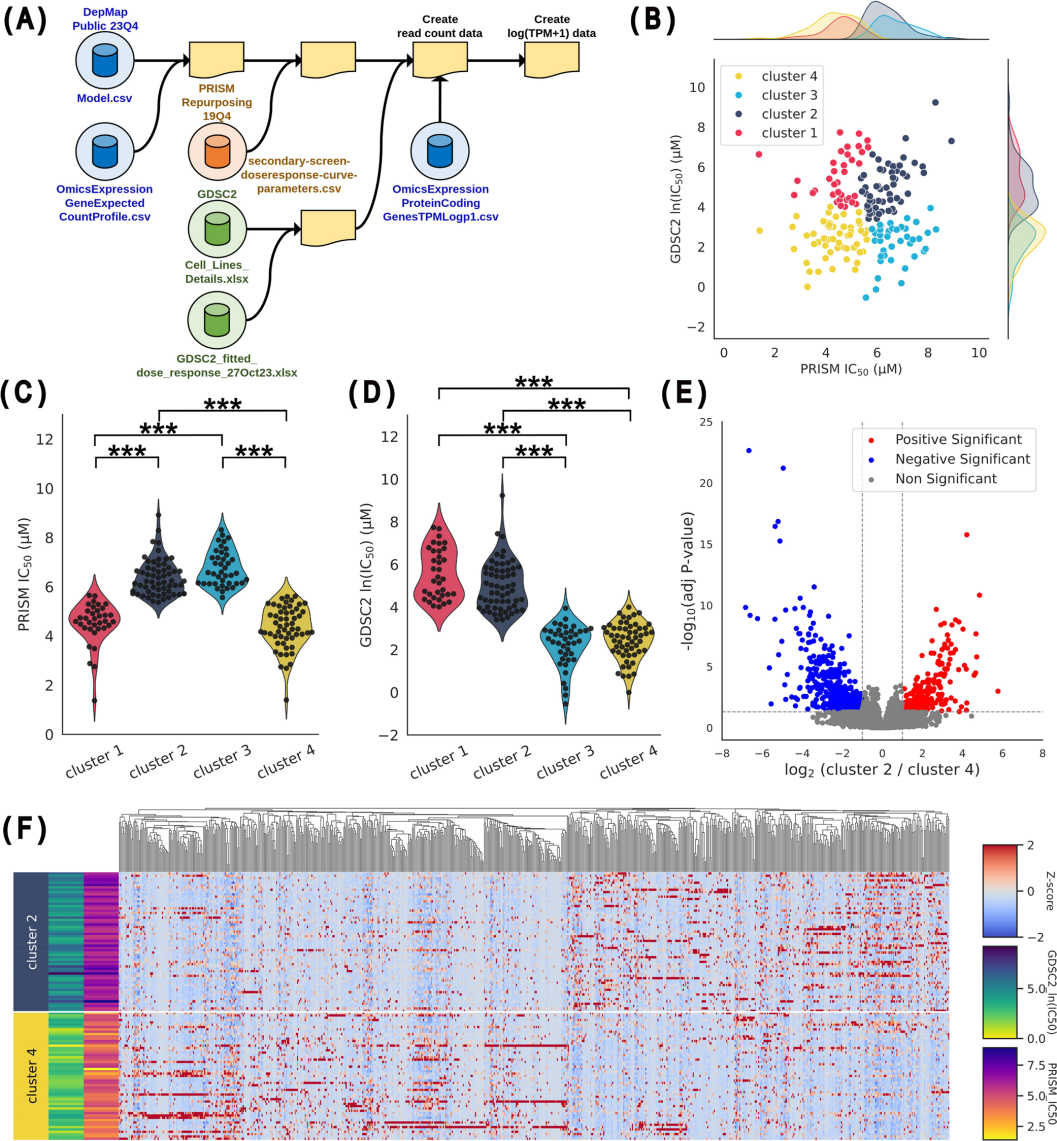
Defining cisplatin‐resistant and cisplatin‐sensitive cell groups via hierarchical clustering based on IC_50_ and DEG analysis. (A) Three datasets were integrated, namely, DepMap RNA‐seq data, DepMap PRISM project IC_50_ data, and GDSC2 IC_50_ data, to define cisplatin‐sensitive and cisplatin‐resistant groups. Cell line information from different databases was consolidated based on matching COSMIC IDs, resulting in a read count matrix for PyDESeq2 analysis and a TPM matrix for machine learning. (B) Hierarchical clustering (Ward's method, cutoff distance = 8) was performed using the two cisplatin IC_50_ data sets to classify samples into four clusters (cluster 1, *n* = 35; cluster 2, *n* = 60; cluster 3, *n* = 40; and cluster 4, *n* = 55), visualized by a scatter plot. (C, D) Comparisons of cisplatin IC_50_ values derived from PRISM (C) or GDSC2 (D) across clusters using one‐way ANOVA revealed significant differences. Significance levels are denoted ***p < 0.001. (E) Volcano plot of DEGs between clusters 2 and 4 was performed using PyDESeq2 on the read count matrix, with significance defined as adjusted *p*‐value < 0.05 and |log_2_FC| > 1. (F) A clustermap was generated to visualize the gene expression profiles of DEGs between clusters 2 and 4, highlighting clear expression patterns across these groups.

IC_50_ values for cisplatin and bulk RNA‐seq data were matched using public databases. Bulk RNA‐seq data were obtained from the Cancer Dependency Map (DepMap), whereas cisplatin IC_50_ values were sourced from both the profiling relative inhibition simultaneously in mixtures (PRISM), a part of the DepMap initiative, and the genomics of drug sensitivity in cancer (GDSC). Cancer cell lines were merged across these datasets using the shared catalog of somatic mutations in cancer (COSMIC) ID to facilitate data integration (Figure 1A). For RNA‐seq analysis, data from the DepMap project were prepared in two count matrices: a read count matrix for DEG analysis and a log_10_(TPM + 1) matrix for machine learning applications. The cisplatin‐resistant or cisplatin‐sensitive cell groups were defined using hierarchical clustering with the Euclidean distance and Ward's method. The clustering performance was evaluated using the silhouette score [18]. based on cisplatin IC_50_ data from both databases.

### 2.2 DEG Analysis

DEG analyses were performed using a count matrix from the DepMap project and PyDESeq2 [19], a Python implementation of an R package DESeq2, after filtering out low‐expression genes. An adjusted *p*‐value threshold of < 0.05 and an absolute log_2_FC threshold of > 1 were set as criteria for significance.

### 2.3 Extracting SHAP Values

To extract SHapley Additive exPlanation (SHAP) values for all genes, LightGBM [20], a gradient‐boosting model, was selected as the base model for SHAP extraction. To retain all genes for analysis, SHAP values were calculated without prior feature reduction. To prevent overfitting during model training, hyperparameters such as early stopping, L1 regularization, and L2 regularization were adjusted. The model parameters were set with a maximum tree depth of 8, a learning rate of 0.1, and area under the curve (AUC) as the evaluation metric. The detailed parameters are listed in Table S1. The generalization performance of the model was evaluated using an independent test dataset. This dataset was generated by splitting the initial dataset into a training dataset for machine learning and a test dataset for evaluation, while preserving the original class balance. To minimize output bias caused by data split patterns, stratified five‐fold cross‐validation was employed and repeated randomly 12 times. Then, the average SHAP values from these iterations were used. Finally, to ensure reproducibility, random state 42, which is widely adopted in machine learning tutorials, was used for data splitting. The model was retrained and tested.

### 2.4 Feature Elimination

To optimize the key genes used to construct the cisplatin sensitivity prediction model, we applied the recursive feature elimination with cross‐validation (RFECV) tool provided in scikit‐learn, a Python machine learning library. Logistic regression was employed as the base classifier using the scikit‐learn implementation with L2 regularization, using C = 1.0 and the “lbfgs” solver, and with the maximum number of iterations set to 1000. The Venn diagram analysis identified 55 common genes that were selected through DEG analysis and SHAP value extraction. These genes were then fitted to the model to obtain standardized logistic regression coefficients. One gene with the smallest absolute standardized logistic coefficient was eliminated per iteration. After each elimination, model performance was evaluated using five‐fold stratified cross‐validation. These processes were repeated until all genes were removed. From a clinical perspective, specificity was chosen as the primary evaluation metric in order to minimize the possibilities of false positive predictions, in which cisplatin‐sensitive cases are incorrectly predicted as resistant cases.

### 2.5 The Cancer Genome Atlas (TCGA) Clinical Validation

To evaluate the robustness of the cisplatin sensitivity prediction model, clinical validation was performed using clinical data obtained from the TCGA portal. The GDC data transfer tool in TCGA was utilized to extract patients with stage IIA–IIIA NSCLC for whom cisplatin‐based combination chemotherapy is recommended as the standard of care according to guidelines from the American Society of Clinical Oncology and other organizations [21]. These patients were selected from TCGA‐LUSC, TCGA‐LUAD, and CDDP_EAGLE‐1, and the analysis was conducted within the scope of open‐access data.

### 2.6 Establishment of a Cisplatin‐Resistant Cell Line, A549CR

Our in‐house stock cell line A549 was confirmed through STR polymorphism analysis. A549 cells were cultured in Dulbecco's modified Eagle medium supplemented with 10% fetal bovine serum at 37°C in a humidified atmosphere of 5% CO₂. A stock solution of cisplatin was prepared at a concentration of 2 mM in phosphate‐buffered saline and stored at −30°C until use. Cisplatin treatments were incubated under 20 μM concentration for an extended period after a stepwise increase in concentration up to a maximum concentration of 20 μM. During this period, the cisplatin‐containing medium was changed approximately every week.

### 2.7 Cell Viability Assay

Parental A549 cells and established A549CR cells were seeded in 96‐well plates (3000 cells/well), treated with serial dilutions of cisplatin, and incubated for 72 h. Then, 0.5 mg/mL MTT was added, and the cells were incubated for 4 h. The formazan crystal was solubilized with 10% sodium dodecyl sulfate, and the OD570 was measured using a microplate reader (Beckman Coulter).

### 2.8 RNA Extraction and Reverse Transcription‐Quantitative Polymerase Chain Reaction (RT‐qPCR)

Total RNA was extracted using the RNeasy mini kit (Qiagen, Hilden, Germany). cDNA was prepared using the ReverTra Ace qPCR RT Master Mix with gDNA Remover (Toyobo, Osaka, Japan). RT‐qPCR was performed using an Eco Real‐Time PCR System (Illumina, CA, USA) and THUNDERBIRD SYBR qPCR Mix (Toyobo). The specific primer pairs are shown in Table S2. The expression level of each target gene was normalized to GAPDH, and the fold change (FC) was calculated using the 2^−ΔΔCt^ method, with parental A549 cells as the reference.

### 2.9 Statistical Analysis

Statistical analyses were performed using Python libraries. One‐way ANOVA was conducted using SciPy and Tukey's multiple testing correction was carried out with statsmodels. Survival analyses were conducted using the lifelines package to estimate Kaplan–Meier curves, perform log‐rank tests, and fit Cox proportional hazards regression models to calculate hazard ratios and 95% CIs, with the Benjamini–Hochberg procedure applied for multiple testing correction. Statistical significance was defined as *p* < 0.05.

## 3 Results

### 3.1 Hierarchical Clustering of Cancer Cell Lines Defined Cisplatin‐Resistant and Cisplatin‐Sensitive Cell Groups

To define cisplatin‐resistant or cisplatin‐sensitive cell groups for building a machine learning model that predicts cisplatin sensitivity, hierarchical clustering was first performed using cisplatin IC_50_ from two publicly available databases (PRISM and GDSC2). The integration of the datasets using COSMIC ID resulted in a dataset including information from 320 cell lines (Figure 1A and Table S3). After excluding samples with missing IC_50_ values or RNA‐seq information, the remaining dataset contained 190 cancer cell lines overlapping in both databases. Next, these cell lines were clustered by unsupervised machine learning to define cisplatin‐sensitive or resistant groups. Silhouette score analyses were performed to determine the optimal number of clusters and showed that the hierarchical clustering method provided four clusters with clear separation (Figure S1A). Whereas the k‐means clustering fragmented the data into nine clusters (Figure S1B), thereby reducing interpretability (Figure S1C–E). Accordingly, we adopted the hierarchical clustering method and obtained four clusters with good separation (Figure 1B and Table S4) using the cluster dendrogram derived from IC_50_ values (Figure S2). Among these clusters, cluster 2 (*n* = 60) and 4 (*n* = 55) exhibited consistent sensitivity profiles between two datasets, while cluster 1 (*n* = 35) and 3 (*n* = 40) did not. Cluster 2 possessing high IC_50_ values in both databases was defined as the cisplatin‐resistant group. In contrast, cluster 4 with low IC_50_ values was defined as the cisplatin‐sensitive group (Figure 1C,D). The cutoff IC_50_ between the sensitive and resistant groups was approximately 30 μM, which was the same level as the maximum plasma concentration of cisplatin in humans (30 μM) [22], indicating that the machine‐driven classification of the cisplatin‐resistant and cisplatin‐sensitive groups through hierarchical clustering is clinically relevant. This analysis identified multiple DEGs between clusters 2 and 4 (Figure 1E and Table S5). A clustermap visualization of gene expression profiles between clusters 2 and 4 confirmed a clear difference across these clusters (Figure 1F). These results imply that the IC_50_‐based clustering successfully classified cancer cells with phenotypes that had differential gene expression profiles.

### 3.2 SHAP Values of Each Gene Were Obtained Through Cross‐Validation of the SHAP Extraction Model

**FIGURE 2:**
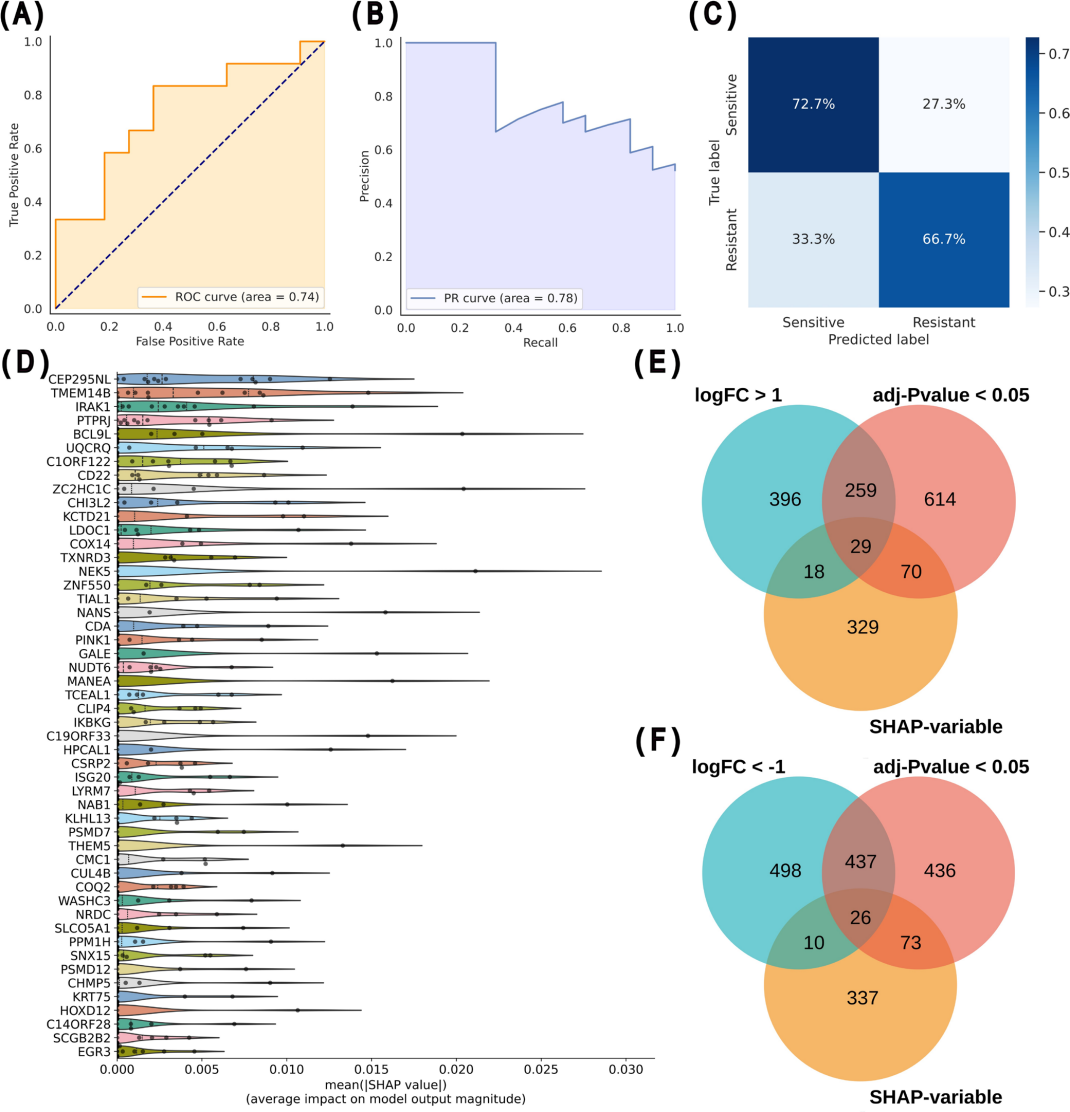
Integration of machine learning for gene contribution analysis into DEG analysis. (A, B) The ROC (A) and the precision–recall (B) curves on the test dataset of the constructed machine learning model for SHAP extraction. (C) A confusion matrix for the test dataset shows the classification performance for sensitive and resistant groups in the SHAP extraction model. (D) Average SHAP values for the top 50 genes across 12 rounds of cross‐validation. Dots represent the SHAP values for each gene in individual cross‐validation. (E, F) Venn diagram displaying 29 genes (E) or 26 genes (F) with positive or negative log_2_FC from DEG analysis respectively and variable SHAP values from machine learning.

To identify the genes of interest, conventional DEG analysis relies on the gene list extracted by solely large changes in relative expression levels [23, 24, 25]. In this study, we employed a machine learning model capable of high‐dimensional RNA‐seq analysis in addition to conventional DEG analysis to integrate gene–gene interactions [26]. A binary classification model for SHAP extraction was built to identify feature genes associated with cisplatin sensitivity, using the resistant and sensitive clusters (clusters 2 and 4, respectively, in Figure 1) as labels and all genes as input features. The contributions of each gene were subsequently analyzed using SHAP values [27, 28]. We evaluated model performance using a stratified test dataset that did not overlap with the training dataset. SHAP values were output using 12 random data splits and 5‐fold cross‐validation. On the test dataset, the evaluation metrics showed an accuracy of 0.70, AUC of 0.74 for the receiver operating characteristics (ROC) curve (Figure 2A), and an AUC of 0.78 for the precision–recall curve (Figure 2B), confirming reliable differentiation between cisplatin‐sensitive and cisplatin‐resistant groups (Figure 2C). In addition, by calculating the average SHAP values obtained from 12 cross‐validations, 447 genes that contributed to the sensitivity predictions were identified (Figure 2D). The SHAP value of each gene is shown in Table S6. Furthermore, to identify cisplatin‐specific biomarker genes, we visualized the overlap of genes between the results of DEG analysis and the output of the machine learning model using a Venn diagram. This analysis identified 29 common genes (Figure 2E) and 26 common genes (Figure 2F) with positive and negative log_2_FC values, respectively.

### 3.3 CSP26G, A Logistic Regression Model, Accurately Predicted Cisplatin Sensitivity

**FIGURE 3:**
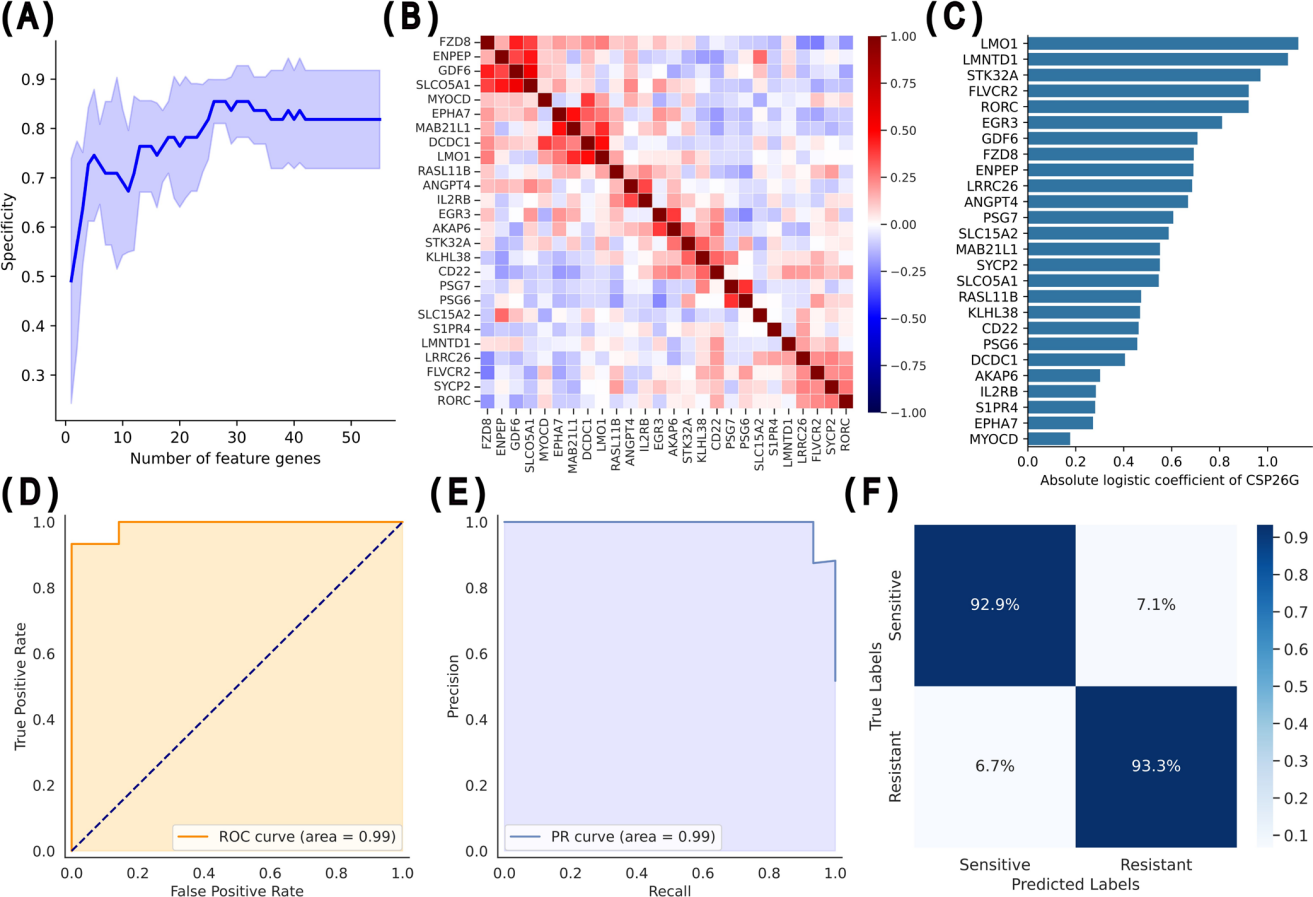
Identification of cisplatin‐specific biomarker gene sets through the feature gene selection and creation of a predictive model for cisplatin sensitivity. (A) Specificity following stepwise elimination of feature genes in ascending order of absolute logistic regression coefficients, visualized with five‐fold cross‐validation. The solid blue line shows the mean specificity from the five‐fold cross‐validation, and the light blue shading indicates the standard deviation, with peak specificity observed at 26 genes. (B) Heatmap of the correlation coefficients among the 26 genes with the highest specificity clustered to show gene–gene relationships. No strong correlation was found among most genes. (C) Absolute values of the logistic regression coefficients for CSP26G. (D, E) Results of CSP26G on the test data. The ROC (D) and the precision–recall (E) curves are illustrated. (F) confusion matrix of CSP26G. These results indicate high discriminatory power between the cisplatin‐resistant and cisplatin‐sensitive groups.

To refine 55 feature genes (29 up‐ and 26 downregulated genes), a logistic regression model was built, and RFECV was applied to select genes with higher contributions based on the absolute regression coefficients. RFECV revealed that 26 genes optimized the model specificity (Figure 3A and Table S7), and correlation analysis of the 26 selected genes revealed that no pairs exhibited strong correlations (Figure 3B). These results imply that each of the 26 genes independently contributes to the high predictive power. The logistic regression coefficients for each of the 26 genes reflect the influence of the gene in the model (Figure 3C). A cisplatin sensitivity predictor using 26 genes (CSP26G) was developed using a scikit‐learn regularized logistic regression model with default hyperparameters, except that the maximum number of iterations was set to 1000. CSP26G demonstrated excellent predictive performance in the stratified dataset, which was independent of the training dataset, with AUC of 0.99 for the ROC curve (Figure 3D), 0.99 AUC for the precision‐recall curve (Figure 3E), and a sensitivity of 0.93 and a specificity of 0.93 (Figure 3F). Furthermore, using the test data employed during model construction, we experimentally evaluated the CSP26G score cutoff value incrementally from 0.1 to 0.9 in 0.1 increments (Figure S3A–I). The results confirmed that the most balanced performance was observed within the range of 0.5–0.7 (Figure S3J). Histogram analysis also confirmed that no outputs exist within this 0.5–0.7 range (Figure S3K).

### 3.4 The CSP26G Was Applicable for External Data

**FIGURE 4:**
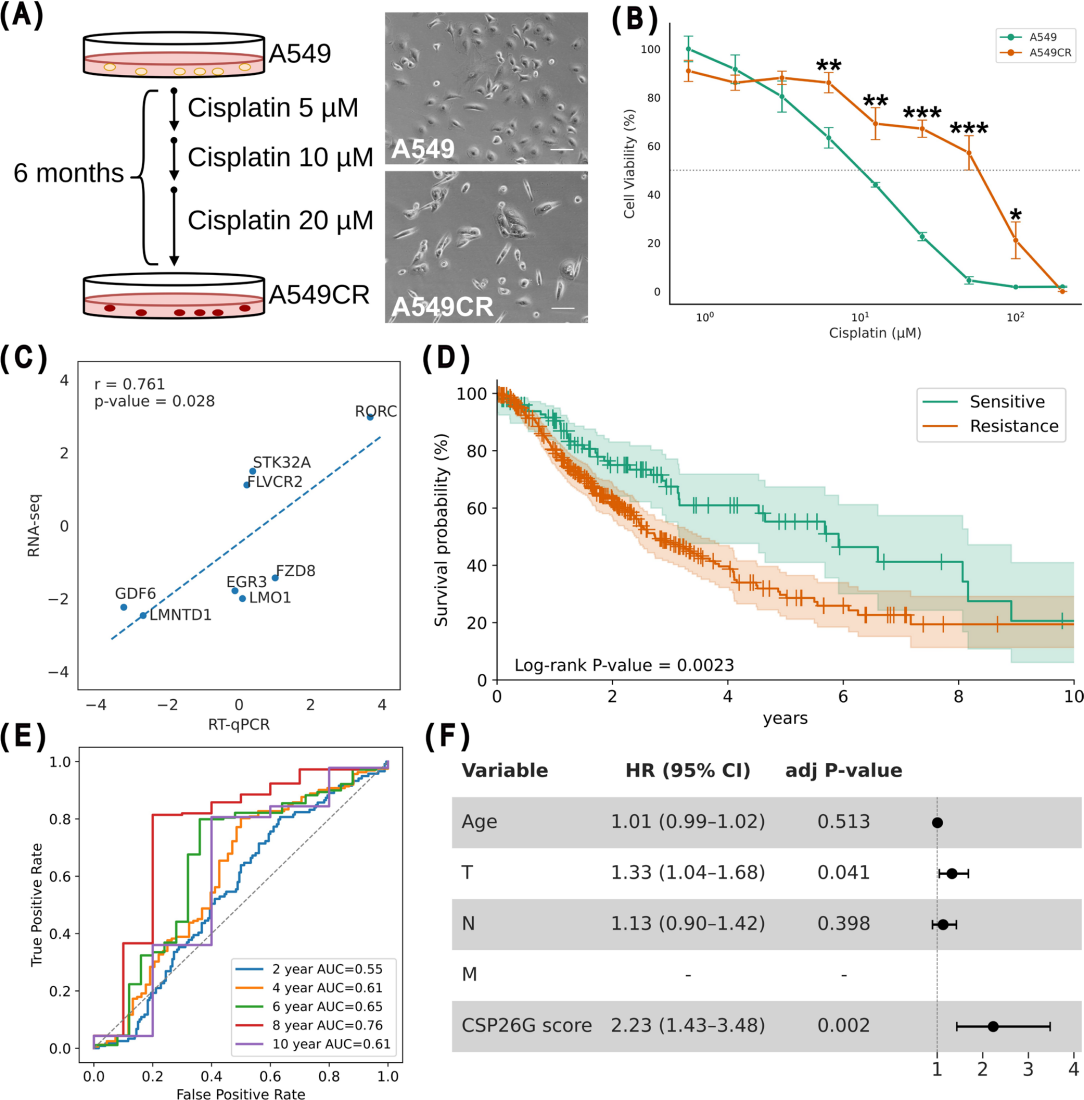
The CSP26G model was applicable for predicting the response to cisplatin in external samples. (A) Schematic representative of A549CR cell establishment and photo images of parental A549 and established A549CR cells are shown. (B) The viabilities of A549 and A549CR cells at 72 h after cisplatin treatment were compared by the MTT assay. Scale bars: 100 μm. Mean ± SD. Significance levels denoted *p < 0.05, **p < 0.01, and ***p < 0.001. (C) RNA‐seq (resistant/sensitive in the training data for CSP26G) and RT‐qPCR (A549CR/A549) showed positive correlation. The x‐ and y‐axes were shown in log_2_ scale, with *r* denoting the Pearson correlation coefficient and its associated *p*‐value. RT‐qPCR data were normalized to GAPDH before fold change calculation. (D) Survival analysis was performed using the Kaplan–Meier method on NSCLC patients with pathological stage II and IIIA (*N* = 407) from TCGA, based on CSP26G output results. The solid green and solid orange lines are the survival curves for the sensitive and resistant groups, respectively. The light green and orange shading on the plot represents the 95% CI for each group. Censored patients are shown as markers on the curve. Statistical analysis was conducted using the log‐rank test. (E) ROC curves at 2, 4, 6, and 8 years of treatment in TCGA data were used to estimate predictive accuracies of CSP26G. (F) Forest plot of multivariate Cox proportional hazards regression for overall survival. Hazard ratios and 95% CI are shown for CSP26G prediction score, age, TNM classifications (T, primary tumor and local invasion; N, regional lymph node involvement; M, distant metastasis). Due to the single level of the M stage, its hazard ratio, 95% CI and *p*‐value could not be calculated.

To evaluate the generalizability of CSP26G, a cisplatin‐resistant cell line, A549CR, was established by continuously exposing the parental A549 to cisplatin for 29 weeks (Figure 4A). An MTT assay revealed that while the IC_50_ of the parental A549 was 10.02 ± 0.77 μM, that of A549CR was 57.53 ± 8.50 μM, indicating a significantly higher viability of A549CR than that of A549 in the presence of cisplatin (Figure 4B). The expressions of genes with high contributions in CSP26G were compared between A549 and A549CR cells by RT‐qPCR. The RT‐qPCR log_2_(A549CR/A549) closely matched the RNA‐seq log_2_(resistant/sensitive) in the training data for CSP26G, with a significantly positive correlation between the RT‐qPCR and RNA‐seq data (Figure 4C). These findings indicate that the resistance‐associated gene expression pattern in CSP26G was recapitulated in externally developed cisplatin‐resistant cells, thereby supporting the validity of the biomarkers identified in this study.

Utility of CSP26G was further validated using clinical data in the TCGA database. CSP26G was applied to tumor‐derived RNA‐seq data from patients with pathological stage IIA–IIIA NSCLC, who generally receive platinum‐based adjuvant chemotherapy [21], and survival analyses were performed based on the model outputs. The survival time of the CSP26G‐predicted resistant group was significantly shorter than that of the predicted sensitive group (median OS: 2.74 vs. 5.92 years, respectively), and the survival curves diverged during the mid‐term follow‐up period, with better survival observed in the group predicted to be sensitive using CSP26G (Figure 4D). Consistent with this pattern, ROC analysis demonstrated peak discriminative ability at 8 years (AUC = 0.76, Figure 4E). Multivariate Cox regression identified the CSP26G score as the strongest independent predictor of survival time, even after adjusting for age, TNM classifications (Figure 4F). Unlike NSCLC stage IIa–IIIa patients who consistently receive cisplatin‐based adjuvant therapy, CSP26G was not predictive in the esophageal and ovarian cohorts where the percentage of patients who received cisplatin is unclear (data not shown). Shifting the decision threshold within the 0.2–0.8 range resulted in only a handful of cases being reassigned to the opposite class (Figure S4A–G). In addition, a histogram of the predicted probability distribution revealed that most values were clustered at both extremes (Figure S4H). These findings indicate that CSP26G‐based stratification showed a clear divergence in survival curves during the mid‐term follow‐up, suggesting its clinical relevance in predicting long‐term recurrence and treatment resistance.

### 3.5 CSP26G Also Predicts Sensitivity to DNA‐Damaging Anticancer Agents Other Than Cisplatin

**FIGURE 5:**
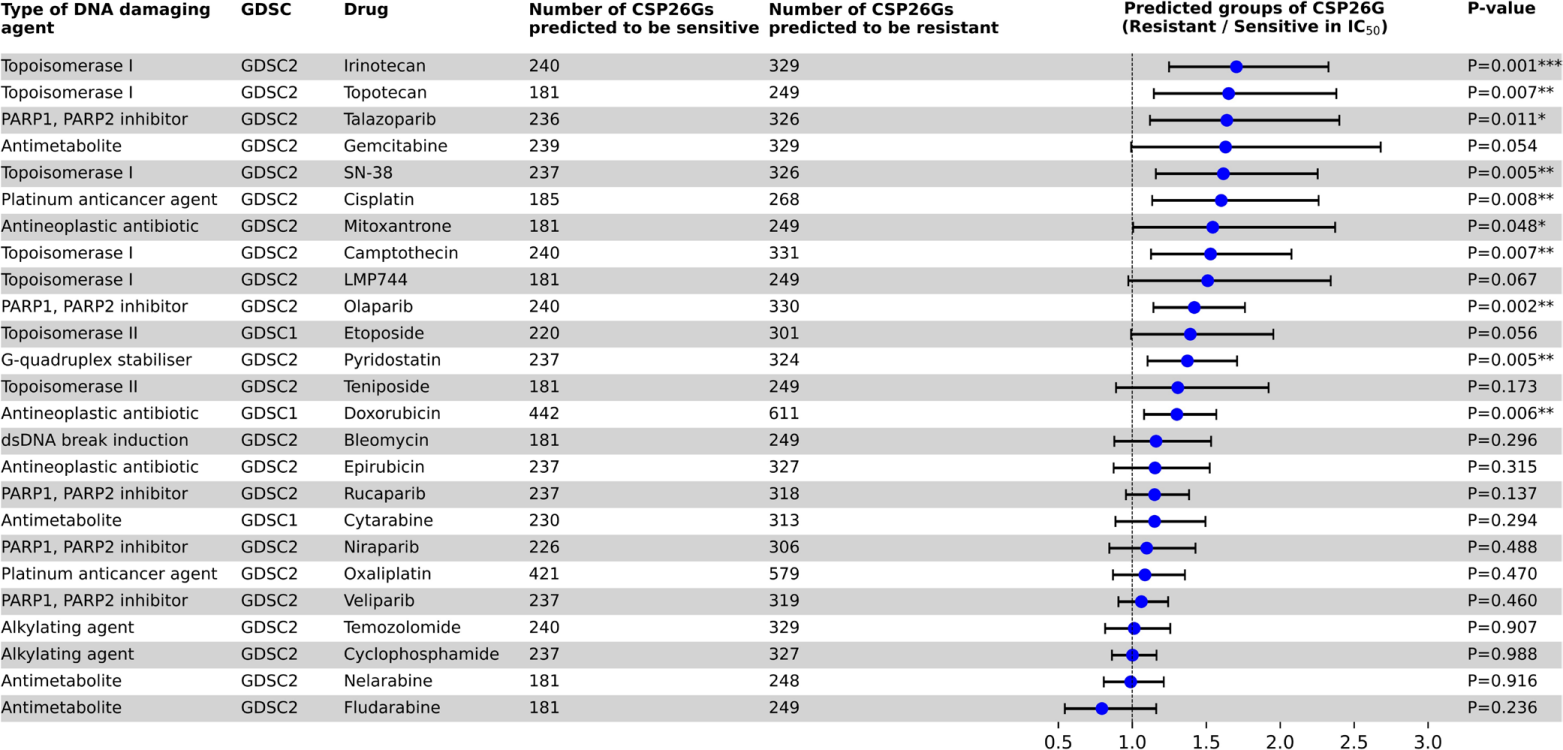
CSP26G may predict the antitumor effects of various DNA‐damaging agents. The left part of the panel lists drug categories in the genomics of drug sensitivity in cancer (GDSC), anticancer agent names, and numbers of cell lines predicted by CSP26G to be sensitive or resistant. The right part shows the fold change in IC_50_ (resistant/sensitive) for each drug, along with its 95% CI. A vertical dashed line at a fold change of 1 indicates no difference, and *p*‐values for each drug are displayed to the right. Fold change values are represented by blue markers, and horizontal bars indicate the CI. Statistical analysis was conducted using Welch's *t*‐test. Significance levels denoted as *p < 0.05, **p < 0.01, and ***p < 0.001.

To evaluate whether CSP26G can predict the efficacy of other anticancer agents as well as cisplatin, cell line data excluding those used for CSP26G development were used. RNA‐seq data for the test cell lines were obtained from DepMap, preprocessed, and then input into CSP26G to predict sensitivity. Then, the IC_50_ values of each cell line to drugs annotated with “DNA replication” in the pathway name of the GDSC database were compared with the CSP26G outputs by evaluating the differences between the sensitive and resistant groups. In the analysis of cisplatin, which was the primary agent of the original objective, the group predicted as resistant by CSP26G exhibited significantly higher IC_50_ values than the group predicted as sensitive, confirming that the model can classify independent test data. In addition to cisplatin, CSP26G successfully predicted the efficacy of topoisomerase I inhibitors, such as irinotecan and camptothecin, and poly (ADP‐ribose) polymerase (PARP) inhibitors, such as olaparib and talazoparib (Figure 5). Interestingly, however, it did not predict the efficacy of oxaliplatin.

## 4 Discussion

This study aimed to improve the prediction of the antitumor efficacy of classical anticancer agents with low specificity. First, we defined cisplatin‐sensitive and cisplatin‐resistant groups using hierarchical clustering. This allowed us to select cell lines with consistent sensitivity profiles regardless of the methodologies employed for data acquisition (traditional cell viability assays in GDSC2 and population measurements using pooled barcode cell lines in PRISM). Then, focusing on clusters 2 and 4, which exhibited robust consistency in sensitivity profiles, conventional bulk RNA‐seq analyses were supplemented with an additional analytical dimension in the form of SHAP values derived from a machine learning model. Finally, we identified the original 26‐gene set that led to the development of the cisplatin sensitivity predictor, CSP26G. CSP26G achieved high scores of 0.93 sensitivity and 0.93 specificity in the test dataset, raising suspicions of overfitting. However, experimental and clinical verification confirmed its high generalization performance, which could not be explained by overfitting. External validation using the A549CR cell line confirmed that the gene expression pattern in A549CR was consistent with that observed in the training data (Figure 4C). Moreover, clinical validation using TCGA data of patients with stage IIA–IIIA NSCLC who received platinum treatment as the standard therapy revealed that patients predicted to be sensitive to cisplatin showed significantly longer survival time than those in patients predicted to be resistant during the mid‐term follow‐up period (Figure 4D), suggesting that CSP26G predicts life‐extending benefit over years, although a larger sample size is needed for more accurate long‐term follow‐up. Notably, CSP26G showed potential to predict not only the efficacy of cisplatin but also those of other DNA‐damaging anticancer agents such as topoisomerase I inhibitors. However, it was unable to predict the efficacy of oxaliplatin, another platinum‐based drug (Figure 5).

Integrating bulk RNA‐seq with machine learning identified a 26‐gene set, and the CSP26G model was developed. Using SHAP values to profile gene expression patterns beyond the reach of conventional DEG analysis (Figure 2D), a unique gene set predictive of cisplatin antitumor efficacy was identified (Table S7), which formed the basis of the CSP26G model (Figure 3D–F). A previous attempt to predict cisplatin efficacy using SHAP values involved individual survival analyses for each extracted gene, and four genes that contributed to efficacy prediction were identified [17]. Although these findings support the utility of machine learning in analyzing high‐dimensional gene networks, those studies did not fully exploit the advantage of incorporating gene–gene interactions. In contrast, the present study examined the overall expression pattern of the 26‐gene set rather than focusing on single genes, thereby enhancing both the predictive accuracy and generalizability of CSP26G compared with a previous model. Collectively, these results demonstrate that combining bulk RNA‐seq and machine learning can facilitate the development of predictive models based on the gene expression patterns reflecting sensitivity to classical anticancer agents.

The 26‐gene set included genes potentially associated with cisplatin resistance. These genes include the tumor suppressor *EPHA7* [29], the apoptosis‐related gene *EGR3* [30], the organic anion transporter *SLCO5A1* [31], which may be involved in drug uptake, and the poorly characterized putative mitotic checkpoint kinase *STK32A* [32]. Interestingly, the gene set also contained multiple genes associated with the regulation of immune responses, such as *RORC* [33] and *S1PR4* [34], even though CSP26G was constructed using cultured cell models in which immunity does not play a direct role. This suggests their potential novel biological relevance that remains to be explored. To further explore the pathways underlying these findings, we performed Gene Ontology (GO) enrichment analysis. However, no statistically significant pathways were identified (data not shown). These results suggest that CSP26G may reflect functional aspects that conventional GO analysis cannot readily capture, possibly due to the small number of genes included in the model or the diversity of underlying mechanisms. One possible reason is that CSP26G was constructed by integrating SHAP values, which were on a different axis than gene expression levels. Previous reports have identified biomarkers such as KRT17P3 [35] and RPS6 [36] and have particularly highlighted SLFN11, which is known to induce TP53‐independent apoptosis, as a predictive marker of DNA‐damaging agent efficacy [37, 38, 39]. Although SLFN11 was not included in the final 26‐gene set, its expression in the cisplatin‐resistant group was approximately 50% of that in the cisplatin‐sensitive group, consistent with previous findings (Table S5). Notably, SHAP analysis revealed that SLFN11 was one of the 446 genes that exhibited significant variation in SHAP values (Table S6).

CSP26G exhibits potential for application not only to cisplatin but also to other anticancer agents. It also demonstrated a predictable capacity for other DNA‐damaging anticancer agents including topoisomerase I and PARP inhibitors (Figure 5). However, CSP26G could not predict the efficacy of oxaliplatin even though it is a platinum anticancer agent. Oxaliplatin was developed to overcome cisplatin resistance [40]. Since its chemical structure differs significantly from that of cisplatin, its mode of DNA interaction is also expected to be different. In fact, the mismatch repair complex is able to recognize cisplatin‐DNA adducts, but not oxaliplatin‐DNA adducts [41, 42]. Moreover, recent studies have indicated that the anticancer activity of oxaliplatin is predominantly driven by nucleolar stress and ribosomal biosynthesis stress, rather than by classical DNA damage [43]. These indicate that platinum‐based anticancer agents are functionally diverse even though they commonly contain platinum in their structure. On the other hand, CSP26G was capable of predicting the efficacy of drugs with mechanisms related to DNA single‐strand breaks, such as cisplatin, topoisomerase I inhibitors, and PARP inhibitors. However, it was difficult to predict the sensitivity of drugs with DNA alkylation as the primary mechanism of action, such as temozolomide and cyclophosphamide (Figure 5). These findings suggest that classification based on chemical structure does not necessarily reflect the mechanism of action or cellular response, and that reclassification based on the cellular response pathways induced by each drug shows a better fit with CSP26G. Thus, this study may become the starting point that integrates common sensitivity prediction indicators and common resistance mechanisms at the pathway level, potentially contributing to the systematization of treatment strategies in clinical settings, such as overcoming resistance and reusing biomarkers.

In summary, CSP26G was developed by the integration of machine learning with bulk RNA‐seq analysis, and it accurately reflects tumor phenotypes and predicts response to standard anticancer agents. Further studies on tumors classified as resistant in CSP26G may contribute to the improvement of recurrent cancer treatment. Notably, compared with the cisplatin‐sensitive group, the cisplatin‐resistant group exhibited significantly lower IC_50_ values for docetaxel, pemetrexed, and palbociclib, suggesting alternative therapeutic options for cisplatin‐resistant malignancies. In addition, preliminary adjustment of the cutoff values using the test dataset at the time of model construction revealed that the range of 0.5–0.7 provided the most balanced model performance (Figure S3J). Therefore, the CSP26G score cutoff was kept at the default setting, but more precise determination and adjustment of the cutoff using clinical datasets is required for clinical application. A limitation of this study is that survival analysis should ideally be conducted using all relevant clinical information. Our future studies will clarify the biological functions and mechanisms of the biomarker genes identified in this study, and this analytical method will be applied to other anticancer agents, contributing to the clinical implementation of precision cancer medicine.

## Author Contributions

**Haruki Ujiie:** conceptualization (equal), data curation (lead), funding acquisition (equal), investigation (lead), methodology (equal), validation (lead), visualization (lead), writing – original draft (equal), writing – review and editing (equal). **Tomoko Sakyo:** investigation (supporting), validation (supporting). **Konomi Oya:** investigation (supporting), validation (supporting). **Yuto Sugawara:** investigation (supporting), validation (supporting). **Miyu Ota:** investigation (supporting), validation (supporting). **Honami Yonezawa:** investigation (supporting). **Naoyuki Nishiya:** conceptualization (equal), funding acquisition (equal), methodology (equal), supervision (lead), writing – original draft (equal), writing – review and editing (equal).

## Ethics Statement

The authors have nothing to report.

## Conflicts of Interest

The authors declare no conflicts of interest.

## Supporting information


**Figure S1:** Silhouette score analyses determined the optimal cluster numbers for hierarchical clustering and k‐means clustering. (A) Hierarchical clustering, which showed the highest silhouette score at four clusters. (B) K‐means clustering, which indicated an optimum at nine clusters. (C) Visualization of the optimal k‐means clustering using a scatter plot. (D) Comparison of cisplatin IC_50_ values across clusters in the PRISM database. (E) Comparison of cisplatin IC_50_ values across clusters in the GDSC2 database.
**Figure S2:** Result of hierarchical clustering. A dendrogram of hierarchical clustering is shown. Using a cutoff distance of 8, 190 cancer cell lines were divided into four clusters.
**Figure S3:** Impact of cutoff value variation on CSP26G performance. (A–I) Confusion matrices illustrating the classification results when the cutoff threshold was varied from 0.1 to 0.9 in increments of 0.1 using the independent test dataset. (J) Line plots of evaluation metrics (Accuracy, Recall, Precision, NPV, Specificity, F1‐score), showing optimal balance at 0.5–0.7. (K) Histogram of CSP26G output scores in the test dataset.
**Figure S4:** Cutoff dependent classification results of CSP26G in the TCGA dataset at 8‐year survival. (A–G) Confusion matrices showing the classification of NSCLC patients when the cutoff threshold of the CSP26G score was varied from 0.2 to 0.8 in increments of 0.1. The ground truth labels were defined based on 8‐year survival status, since this point yielded the highest performance in ROC analysis. (H) Distribution of CSP26G output scores among TCGA NSCLC patients.

**Table S1:** LightGBM hyperparameter settings used for SHAP value extraction and model training.
**Table S2:** Summary of RT‐qPCR primer sequence.
**Table S3:** List of cell lines.
**Table S4:** Hierarchical clustering analysis of cisplatin sensitivity.
**Table S5:** Results of DEG analysis between cluster 2 (Resistant) and cluster 4 (Sensitive) using pyDESeq2.
**Table S6:** List of SHAP values for each gene based on machine learning model.
**Table S7:** Overview of the cisplatin‐specific 26 gene sets.

## Data Availability

Raw data supporting this study's findings were obtained from publicly accessible databases, including the DepMap Project (https://depmap.org/portal/), PRISM Repurposing Dataset (https://depmap.org/repurposing/), The Cancer Genome Atlas (https://portal.gdc.cancer.gov/), and the Genomics of Drug Sensitivity in Cancer (https://www.cancerrxgene.org/downloads). Tables S3, S5 and S6 generated in this study have been deposited in Zenodo (DOI: https://doi.org/10.5281/zenodo.16786026). Processed data supporting additional findings are available in the Supporting Information of this article.
