# Supplementary material for: Machine Learning–Driven Integration of Cancer Cell Phenotypes Predicts Cisplatin Sensitivity
Source: Cancer Med. 2025 Nov 20;14(22):e71373. doi: 10.1002/cam4.71373 (PMC12631745; doi:10.1002/cam4.71373)
Supplement: Supplementary file 1 — Figure S1: Silhouette score analyses determined the optimal cluster numbers for hierarchical clustering and k‐means clustering. (A) Hierarchical clustering, which showed the highest silhouette score at four clusters. (B) K‐means clustering, which indicated an optimum at nine clusters. (C) Visualization of the optimal k‐means clustering using a scatter plot. (D) Comparison of cisplatin IC50 values across clusters in the PRISM database. (E) Comparison of cisplatin IC50 values across clusters in the GDSC2 database. Figure S2: Result of hierarchical clustering. A dendrogram of hierarchical clustering is shown. Using a cutoff distance of 8, 190 cancer cell lines were divided into four clusters. Figure S3: Impact of cutoff value variation on CSP26G performance. (A–I) Confusion matrices illustrating the classification results when the cutoff threshold was varied from 0.1 to 0.9 in increments of 0.1 using the independent test dataset. (J) Line plots of evaluation metrics (Accuracy, Recall, Precision, NPV, Specificity, F1‐score), showing optimal balance at 0.5–0.7. (K) Histogram of CSP26G output scores in the test dataset. Figure S4: Cutoff dependent classification results of CSP26G in the TCGA dataset at 8‐year survival. (A–G) Confusion matrices showing the classification of NSCLC patients when the cutoff threshold of the CSP26G score was varied from 0.2 to 0.8 in increments of 0.1. The ground truth labels were defined based on 8‐year survival status, since this point yielded the highest performance in ROC analysis. (H) Distribution of CSP26G output scores among TCGA NSCLC patients. [file CAM4-14-e71373-s002.pdf]

Supplementary Figures

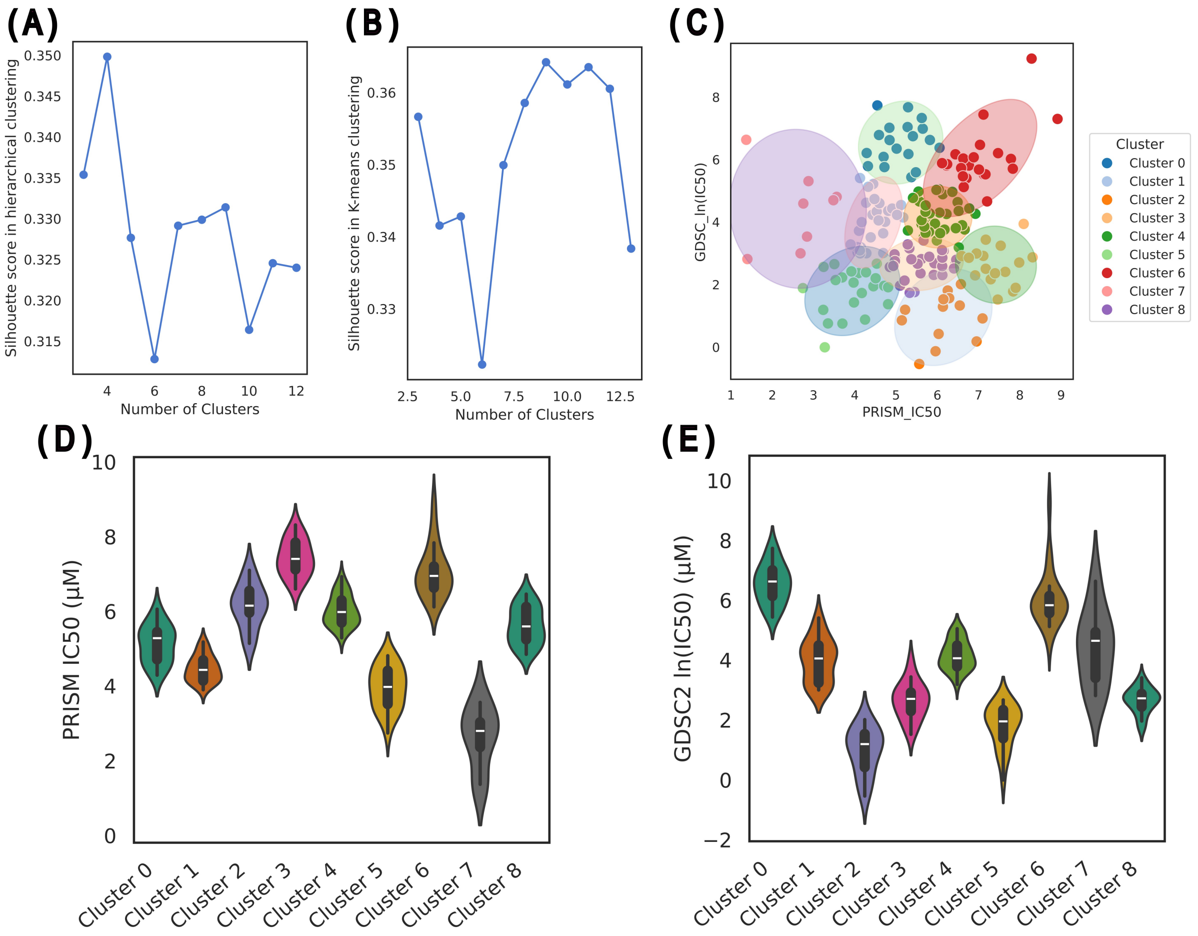

Figure S1 Silhouette score analyses determined the optimal cluster numbers for hierarchical clustering and k-means clustering. (A) Hierarchical clustering, which showed the highest silhouette score at four clusters. (B) K-means clustering, which indicated an optimum at nine clusters. (C) Visualization of the optimal k-means clustering using a scatter plot. (D) Comparison of cisplatin IC50 values across clusters in the PRISM database. (E) Comparison of cisplatin IC50 values across clusters in the GDSC2 database.

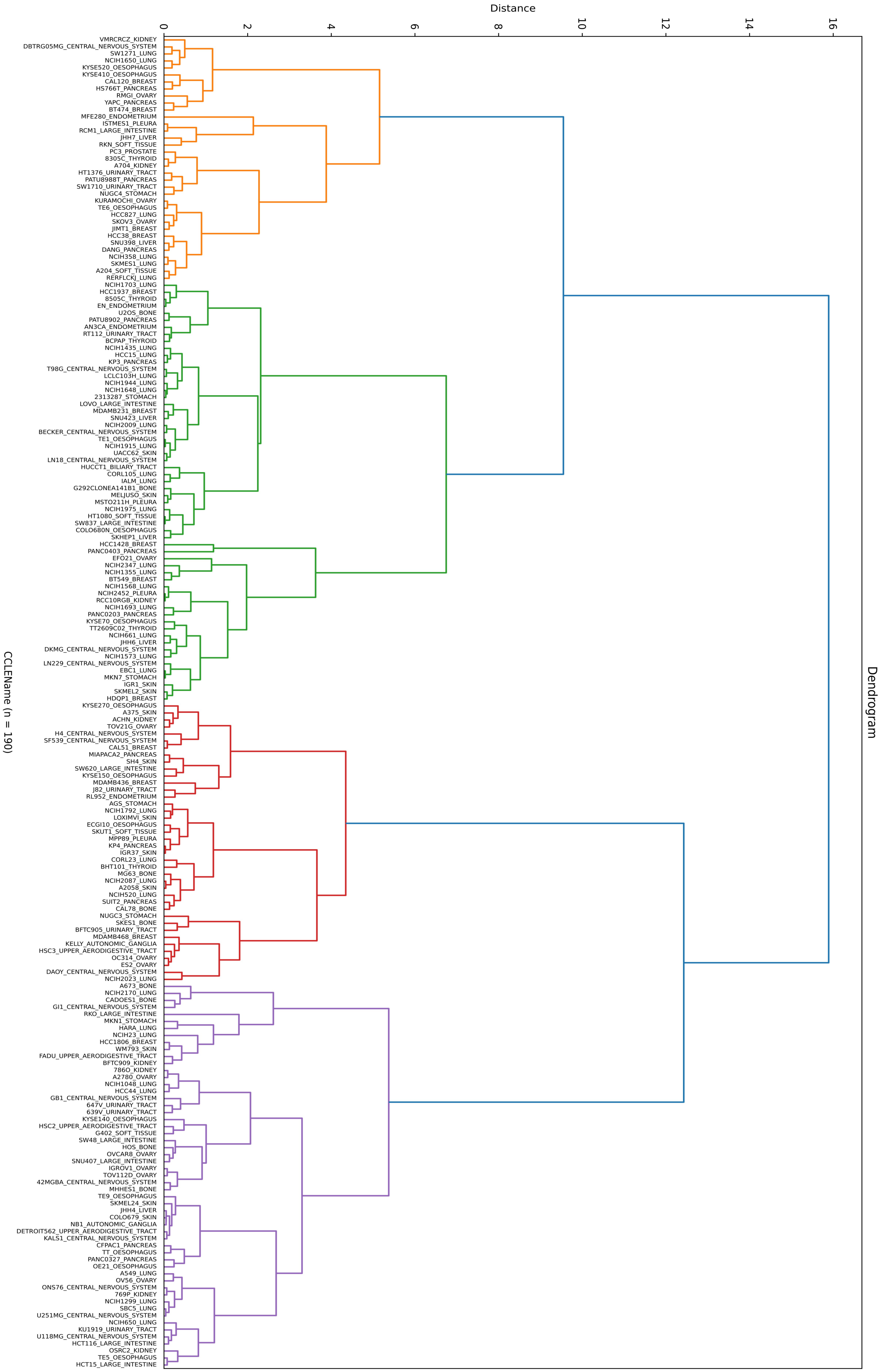

Figure S2 Result of hierarchical clustering. A dendrogram of hierarchical clustering is shown. Using a cutoff distance of 8, 190 cancer cell lines were divided into four clusters.

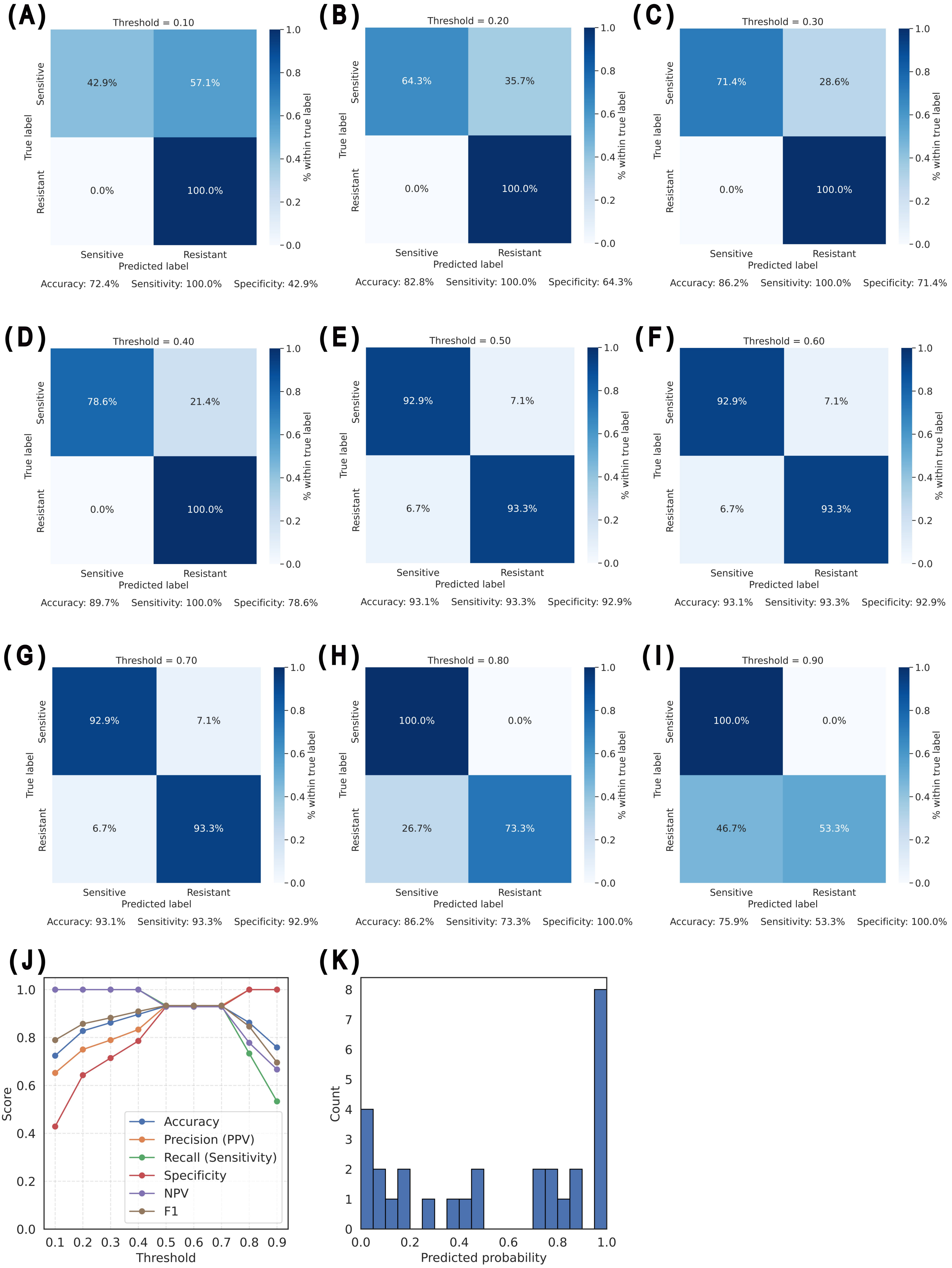

Figure S3 Impact of cutoff value variation on CSP26G performance. (A–I) Confusion matrices illustrating the classification results when the cutoff threshold was varied from 0.1 to 0.9 in increments of 0.1 using the independent test dataset. (J) Line plots of evaluation metrics (Accuracy, Recall, Precision, NPV, Specificity, F1-score), showing optimal balance at 0.5–0.7. (K) Histogram of CSP26G output scores in the test dataset.

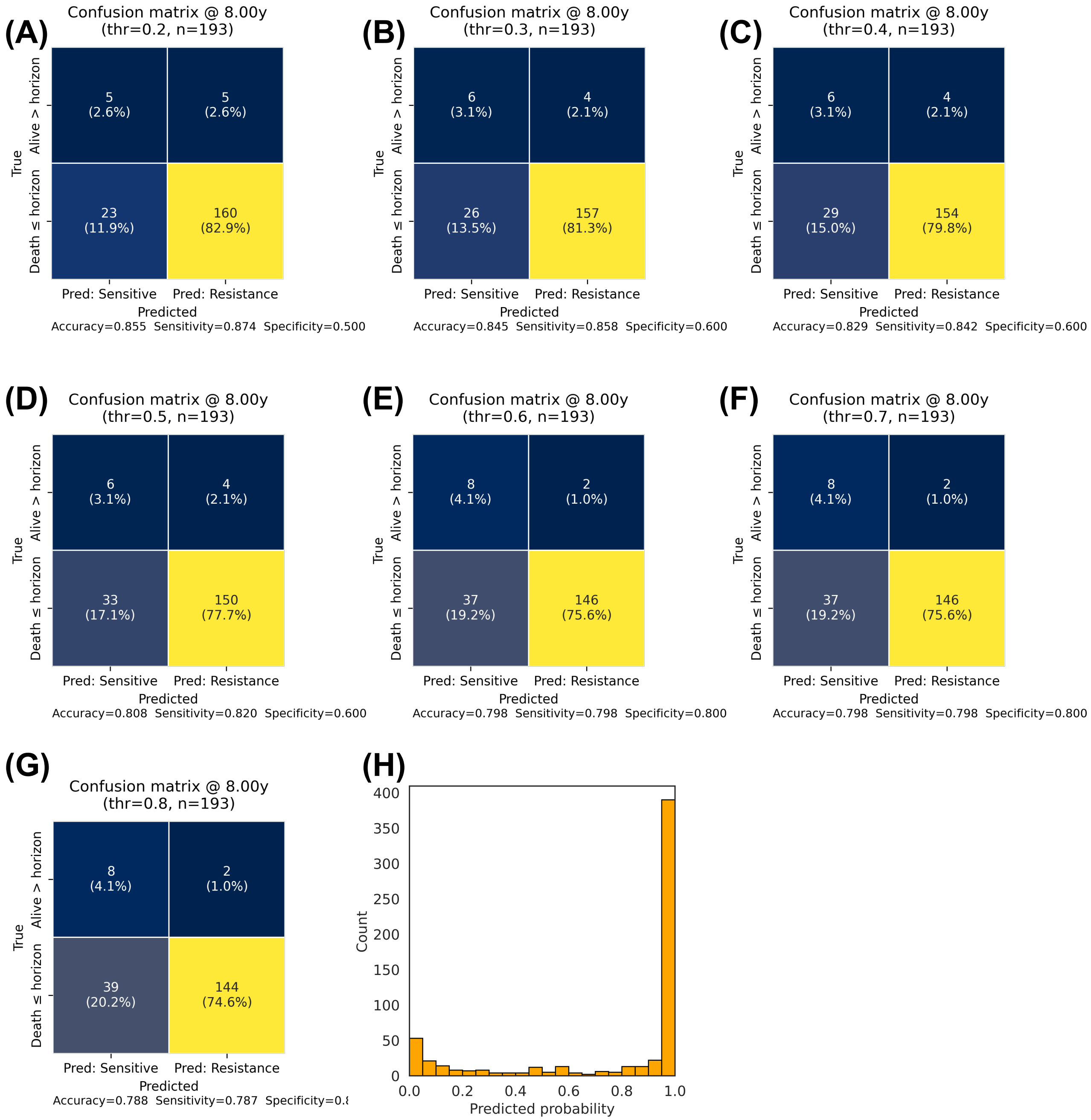

Figure S4 Cutoff dependent classification results of CSP26G in the TCGA dataset at 8 year survival. (A–G) Confusion matrices showing the classification of NSCLC patients when the cutoff threshold of the CSP26G score was varied from 0.2 to 0.8 in increments of 0.1. The ground truth labels were defined based on 8-year survival status, since this point yielded the highest performance in ROC analysis. (H) Distribution of CSP26G output scores among TCGA NSCLC patients.
