# Supplementary material for: Machine Learning–Driven Integration of Cancer Cell Phenotypes Predicts Cisplatin Sensitivity
Source: Cancer Med. 2025 Nov 20;14(22):e71373. doi: 10.1002/cam4.71373 (PMC12631745; doi:10.1002/cam4.71373)
Supplement: Supplementary file 2 — Table S1: LightGBM hyperparameter settings used for SHAP value extraction and model training. Table S2: Summary of RT‐qPCR primer sequence. Table S3: List of cell lines. Table S4: Hierarchical clustering analysis of cisplatin sensitivity. Table S5: Results of DEG analysis between cluster 2 (Resistant) and cluster 4 (Sensitive) using pyDESeq2. Table S6: List of SHAP values for each gene based on machine learning model. Table S7: Overview of the cisplatin‐specific 26 gene sets. [file CAM4-14-e71373-s001.zip › Supplementary Tables S1-S7.docx]

**Supplementary Tables**

**Table S1. LightGBM hyperparameter settings used for SHAP value extraction and model training.**

| **Hyperparameter** | **Value** |
| --- | --- |
| n_estimators | 5000 |
| max_depth | -8 |
| learning_rate | 0.1 |
| min_child_weight | 0.1 |
| reg_lambda | 0.1 |
| reg_alpha | 0.4 |
| num_leaves | 93 |
| min_child_samples | 20 |
| subsample | 0.7 |
| colsample_bytree | 0.4 |
| scale_pos_weight | 55/60 |
| subsample_freq | 8 |
| boosting_type | gbdt |
| objective | binary |
| metric | auc |

**Table S2. Summary of RT-qPCR primer sequence.**

| **Target genes** | **Forward primer（5' - 3'）** | **Reverse primer（5' - 3'）** |
| --- | --- | --- |
| GAPDH | GTGGTCTCCTCTGACTTCAAC | GTAGCCAAATTCGTTGTCATACC |
| STK32A | ATGGGAGCGAACACTTCAAGA | CCCTGCATGATCTGGAGTTCC |
| FLVCR2 | CCCTGAGCTATGCCTTGACC | ATCACCATGCGATTCAGAAGAG |
| RORC | CCAAGGCAGGGCTCAATG | GAAGTCCACATCGGTCAGG |
| FZD8 | GCTCTACAACCGCGTCAAGA | GCTGAAAAAGGGGTTGTGGC |
| GDF6 | CAGTCTTCCAAGTCGGCTAATAC | CTGAGAGCATGGACACATCAA |
| EGR3 | GACATCGGTCTGACCAACGAG | GGCGAACTTTCCCAAGTAGGT |
| LMNTD1 | TCCGAAAGCGTGTGTTTCAGT | CTTGAGCTTGTTCCACCTGAT |
| LMO1 | TCTACACCAAGGCCAACCTC | AGCAGTCGAGGTGATACACG |

**Table S3. List of cell lines.**

Due to file size, this table is provided as an external Excel file on <https://doi.org/10.5281/zenodo.16786026>

**Table S4. Hierarchical clustering analysis of cisplatin sensitivity.**

| **OncotreePrimaryDisease** | **cluster 1**  **(n = 35)** | **cluster 2**  **(n = 60)** | **cluster 3**  **(n = 40)** | **cluster 4**  **(n = 55)** |
| --- | --- | --- | --- | --- |
| Adenosquamous Carcinoma of the Pancreas | 0 | 1 | 0 | 0 |
| Anaplastic Thyroid Cancer | 1 | 1 | 1 | 0 |
| Bladder Urothelial Carcinoma | 2 | 1 | 2 | 2 |
| Breast Ductal Carcinoma In Situ | 0 | 0 | 0 | 1 |
| Chondrosarcoma | 0 | 0 | 1 | 0 |
| Colorectal Adenocarcinoma | 1 | 2 | 1 | 5 |
| Diffuse Glioma | 1 | 5 | 2 | 6 |
| Embryonal Tumor | 0 | 0 | 1 | 1 |
| Endometrial Carcinoma | 1 | 2 | 1 | 0 |
| Esophageal Squamous Cell Carcinoma | 3 | 3 | 3 | 5 |
| Esophagogastric Adenocarcinoma | 1 | 2 | 2 | 1 |
| Ewing Sarcoma | 0 | 0 | 1 | 3 |
| Fibrosarcoma | 0 | 1 | 0 | 0 |
| Head and Neck Squamous Cell Carcinoma | 0 | 0 | 1 | 3 |
| Hepatocellular Carcinoma | 2 | 3 | 0 | 1 |
| Intraductal Papillary Neoplasm of the Bile Duct | 0 | 1 | 0 | 0 |
| Invasive Breast Carcinoma | 4 | 5 | 3 | 0 |
| Leiomyosarcoma | 1 | 0 | 0 | 0 |
| Lung Neuroendocrine Tumor | 1 | 0 | 0 | 2 |
| Melanoma | 0 | 4 | 5 | 3 |
| Neuroblastoma | 0 | 0 | 1 | 1 |
| Non-Small Cell Lung Cancer | 5 | 18 | 5 | 7 |
| Osteosarcoma | 0 | 2 | 1 | 1 |
| Ovarian Epithelial Tumor | 3 | 1 | 3 | 5 |
| Pancreatic Adenocarcinoma | 4 | 3 | 3 | 2 |
| Pleural Mesothelioma | 1 | 2 | 1 | 0 |
| Poorly Differentiated Thyroid Cancer | 0 | 1 | 0 | 0 |
| Prostate Adenocarcinoma | 1 | 0 | 0 | 0 |
| Renal Cell Carcinoma | 2 | 1 | 1 | 5 |
| Rhabdoid Cancer | 1 | 0 | 0 | 0 |
| Urethral Cancer | 0 | 0 | 0 | 1 |
| Uterine Sarcoma/Mesenchymal | 0 | 0 | 1 | 0 |
| Well-Differentiated Thyroid Cancer | 0 | 1 | 0 | 0 |

**Table S5. Results of DEG analysis between cluster 2 (Resistant) and cluster 4 (Sensitive) using PyDESeq2.**

Due to file size, this table is provided as an external Excel file on <https://doi.org/10.5281/zenodo.16786026>

**Table S6. List of SHAP values for each gene based on machine learning model.**

Due to file size, this table is provided as an external Excel file on <https://doi.org/10.5281/zenodo.16786026>

**Table S7. Overview of the cisplatin-specific 26 gene sets.**

| **Official Symbol** | **Official Full Name** | **Location** | **Gene Ontology (Function)** | **Gene Ontology (Process)** |
| --- | --- | --- | --- | --- |
| ANGPT4 | angiopoietin 4 | 20p13 | enables receptor tyrosine kinase binding, enables transmembrane receptor protein tyrosine kinase activator activity | NOT involved_in endothelial cell proliferation, involved_in negative regulation of angiogenesis, involved_in negative regulation of apoptotic process |
| S1PR4 | sphingosine-1-phosphate receptor 4 | 19p13.3 | enables lipid binding, enables protein binding | involved_in G protein-coupled receptor signaling pathway |
| MYOCD | myocardin | 17p12 | enables DNA-binding transcription factor binding, enables transcription coactivator activity, enables protein binding | involved_in negative regulation of cyclin-dependent protein serine/threonine kinase activity, involved_in negative regulation of cell population proliferation |
| KLHL38 | kelch like family member 38 | 8q24.13 | enables protein binding, enables ubiquitin-like ligase-substrate adaptor activity | involved_in proteasome-mediated ubiquitin-dependent protein catabolic process |
| LRRC26 | leucine rich repeat containing 26 | 9q34.3 | enables potassium channel activator activity, enables transmembrane transporter binding, enables voltage-gated potassium channel activity | involved_in positive regulation of voltage-gated potassium channel activity, involved_in potassium ion transmembrane transport |
| SYCP2 | synaptonemal complex protein 2 | 20q13.33 | enables DNA binding | involved_in synaptonemal complex assembly |
| PSG6 | pregnancy specific beta-1-glycoprotein 6 | 19q13.31 | - | involved_in female pregnancy |
| CD22 | CD22 molecule | 19q13.12 | enables IgM binding, enables protein binding, enables protein phosphatase binding | involved_in negative regulation of B cell receptor signaling pathway, involved_in regulation of endocytosis |
| RORC | RAR related orphan receptor C | 1q21.3 | enables sequence-specific double-stranded DNA binding, enables DNA-binding transcription repressor activity, RNA polymerase II-specific, enables RNA polymerase II cis-regulatory region sequence-specific DNA binding | involved_in negative regulation of transcription by RNA polymerase II, involved_in cellular response to sterol |
| FLVCR2 | FLVCR choline and putative heme transporter 2 | 14q24.3 | enables heme binding, enables heme transmembrane transporter activity | involved_in choline transport |
| SLC15A2 | solute carrier family 15 member 2 | 3q13.33 | enables dipeptide transmembrane transporter activity, enables peptide:proton symporter activity | involved_in dipeptide import across plasma membrane, nvolved_in antibacterial innate immune response, involved_in xenobiotic detoxification by transmembrane export across the plasma membrane |
| LMNTD1 | lamin tail domain containing 1 | 12p12.1 | - | involved_in cell population proliferation |
| EPHA7 | EPH receptor A7 | 6q16.1 | enables protein binding | involved_in ephrin receptor signaling pathway |
| EGR3 | early growth response 3 | 8p21.3 | enables DNA-binding transcription factor activity, enables sequence-specific double-stranded DNA binding | involved_in cell migration involved in sprouting angiogenesis, involved_in cellular response to vascular endothelial growth factor stimulus, involved_in negative regulation of apoptotic process |
| DCDC1 | doublecortin domain containing 1 | 11p14.1-p13 | enables microtubule binding, enables protein binding | involved_in regulation of mitotic cytokinesis |
| ENPEP | glutamyl aminopeptidase | 4q25 | enables aminopeptidase activity, enables metalloaminopeptidase activity | acts_upstream_of_or_within cell migration, acts_upstream_of_or_within cell population proliferation, involved_in cell-cell signaling |
| RASL11B | RAS like family 11 member B | 4q12 | enables protein binding | involved_in negative regulation of transforming growth factor beta receptor signaling pathway |
| FZD8 | frizzled class receptor 8 | 10p11.21 | enables PDZ domain binding, enables Wnt receptor activity, enables ubiquitin protein ligase binding | involved_in canonical Wnt signaling pathway |
| SLCO5A1 | solute carrier organic anion transporter family member 5A1 | 8q13.3 | enables sodium-independent organic anion transmembrane transporter activity | involved_in sodium-independent organic anion transport |
| LMO1 | LIM domain only 1 | 11p15.4 | enables protein binding | involved_in positive regulation of transcription by RNA polymerase II |
| MAB21L1 | mab-21 like 1 | 13q13.3 | enables protein binding | involved_in anatomical structure morphogenesis, involved_in eye development |
| GDF6 | growth differentiation factor 6 | 8q22.1 | enables protein binding | involved_in BMP signaling pathway, involved_in positive regulation of DNA-templated transcription, involved_in positive regulation of SMAD protein signal transduction |
| AKAP6 | A-kinase anchoring protein 6 | 14q12 | enables protein binding, enables protein-membrane adaptor activity, enables transmembrane transporter binding | involved_in adenylate cyclase-activating G protein-coupled receptor signaling pathway, involved_in positive regulation of cell growth, involved_in positive regulation of potassium ion transmembrane transport |
| IL2RB | interleukin 2 receptor subunit beta | 22q12.3 | enables interleukin-15 receptor activity, contributes_to interleukin-2 receptor activity | involved_in interleukin-15-mediated signaling pathway, involved_in interleukin-2-mediated signaling pathway |
| STK32A | serine/threonine kinase 32A | 5q32 | enables protein binding, enables DNA-dependent protein kinase activity | involved_in chromatin remodeling, involved_in intracellular signal transduction |
| PSG7 | pregnancy specific beta-1-glycoprotein 7 | 19q13.31 | - | involved_in female pregnancy |
